# Evaluation of the Accuracy of Standardized Uptake Values of ^18^F-fluorodeoxyglucose in Lung Lesions Based on Phantom Studies

**DOI:** 10.17691/stm2021.13.3.02

**Published:** 2021-06-28

**Authors:** M.S. Tlostanova, L.A. Chipiga

**Affiliations:** Leading Researcher, Department of Radiation Diagnostics; A.M. Granov Russian Research Center for Radiology and Surgical Technologies, Ministry of Health of the Russian Federation, 70 Leningradskaya St., Saint Petersburg, Pesochniy pos., 197758, Russia; Physician-Radiologist, Department of Radioisotope Positron Emission Tomography; A.M. Granov Russian Research Center for Radiology and Surgical Technologies, Ministry of Health of the Russian Federation, 70 Leningradskaya St., Saint Petersburg, Pesochniy pos., 197758, Russia; Researcher, Laboratory of Radiation Hygiene of Medical Facilities; Saint Petersburg Research Institute of Radiation Hygiene named after Professor P.V. Ramzaev, 8 Mira St., Saint Petersburg, 197101, Russia; Researcher; A.M. Granov Russian Research Center for Radiology and Surgical Technologies, Ministry of Health of the Russian Federation, 70 Leningradskaya St., Saint Petersburg, Pesochniy pos., 197758, Russia; Associate Professor, Department of Nuclear Medicine and Radiation Technologies, Almazov National Medical Research Centre, Ministry of Health of the Russian Federation, 2 Akkuratova St., Saint Petersburg, 197341, Russia

**Keywords:** PET/CT, 18F-FDG, lung lesions, standardized uptake values, recovery coefficients, partial volume effect, NEMA IEC PET Body Phantom Set

## Abstract

**The aim of the study** was to estimate the accuracy of standardized uptake values of ^18^F-fluorodeoxyglucose (^18^F-FDG) in lung lesions during positron emission tomography combined with computed tomography (PET/CT) imaging, based on phantom studies performed for different PET/CT scanners.

**Materials and Methods:**

The analysis of the PET/CT with ^18^F-FDG data was performed for 86 patients newly diagnosed with the lung lesions: malignant tumors (n=37), benign tumors and inflammatory diseases (n=49). The criteria for inclusion in the study were developed considering the recommendations of the Fleischner Society (2017). The characteristics of the lesions on CT met the following requirements: a round shape or close to it; total size of 8 to 30 mm; solid or subsolid structure (with the exception of lesion with ground-glass opacity); a solid part size of ≥8 mm. All the patients had no signs of pleurisy, lymphadenopathy, or cancer history. PET/CT imaging with ^18^F-FDG was performed with three scanners: Discovery 690 (General Electric, USA), Biograph mCT 128 (Siemens, Germany), and Biograph mCT 40 (Siemens); the preparation of patients prior to the scan was standardized. To determine the reference accumulation of a radiopharmaceutical in the pathological lesion, four scans of a specialized NEMA IEC PET Body Phantom Set (USA) were performed for each scanner. For each unit, the recovery coefficients (RCs) of radioactivity, maximum and recovered (corrected) standardized uptake values (SUVs) were determined. Statistical relationship between the size of lesions, SUV_max_ and SUV_correct_ was evaluated. Data processing was performed using MedCalc v. 19.2.0 software.

**Results:**

During the phantom study, the underestimation of the radioactivity was determined in the spheres with the diameters of 10 and 13 mm, overestimation was observed in the sphere with the diameter of 28 mm. Both underestimation and overestimation of radioactivity were determined for the spheres with a diameter of 17 and 22 mm.

SUV_max_ differed from the reference values for 85 patients (98.8%). The underestimation of these values was found for 63 patients (73.2%) due to the partial volume effect. The greatest underestimation was observed for the patients with 8 mm diameter lesions. Depending on the scanner, the underestimation of the SUV_max_ in these patients reached up to 54–73%. For 9 patients (25%) with malignant tumors of 9–12 mm, the utility of RC made it possible to avoid false negative results. For the lesions with a diameter of 30 mm, an overestimation of SUV_max_ up to 22% was determined due to the negative influence of the reconstruction algorithms.

**Conclusion:**

The use of RC eliminates the influence of the partial volume effect and reconstruction methods on the accuracy of estimating the SUV_max_ in lung lesions, which ensures reproducibility, increase in the information content of the method, as well as the comparability of the results of PET/CT with ^18^F-FDG obtained on the different models of PET/CT units with different technological characteristics.

## Introduction

Positron emission tomography (PET) is based on obtaining information about the biodistribution of a radiopharmaceutical in the patient’s body. The accuracy of the evaluation of the radiopharmaceutical accumulation depends on the features of the detecting system of the scanner, radionuclide used, scanning protocol, data reconstruction algorithm, size of a pathological lesion, reconstruction and processing methods, etc [1]. According to the studies [1–4], the use of some data reconstruction methods with the time-of-flight (ToF) technology and point spread function (PSF) leads to overestimation of the standardized uptake values (SUVs). At the same time, an underestimation of the SUV_max_ for small lesions is associated with the partial volume effect (PVE) [5, 6].

The PVE concept combines two related phenomena that negatively affect both the qualitative characteristics of the images and the semi-quantitative values obtained during positron emission tomography combined with computed tomography (PET/CT) [5–7]. The first phenomenon is associated with the existing limitations of the spatial resolution of the PET modality and, as a consequence, blurring of the boundaries of the studied object on a three-dimensional image. This is due to the radioactivity “spill-over” and “spill-in” effects or, in other words, signal displacement from the focus to the surrounding tissues. Since a part of the detected signal becomes visible in the image outside the actual source, the PET images show the sizes of small tumors to seem significantly larger than real size. The second phenomenon is due to the presence of a tissue fraction. It consists of summing and subsequent averaging of the intensity of the detected signal from the tumor focus and nearby tissues, which leads to an artificial underestimation of SUV_max_. This entails the underestimation of the biological aggressiveness of the tumor and, as a consequence, an increase in the number of false negative results.

In other countries, one of the ways to correct PVE is the use of the recovery coefficient (RC), which is determined by scanning a specialized phantom of the NEMA IEC PET Body Phantom Set (USA), recommended by the National Electrical Manufacturers Association (NEMA) [8–10]. This procedure is performed on the ongoing basis, as a part of quality assurance. In the Russian Federation, it is advisory [11]. Meanwhile, the widespread use of RC could improve the accuracy of the method, as well as reproducibility and comparability of the results obtained in medical facilities on PET/CT scanners of various manufacturers with different technical characteristics.

**The aim of this study** was to estimate the accuracy of the evaluation of the standardized uptake values of ^18^F-fluorodeoxyglucose (^18^F-FDG) in lung lesions when performing positron emission tomography combined with computed tomography (PET/CT), based on phantom studies performed for different PET/CT scanners.

## Materials and Methods

### Phantom studies

**Figure 1:**
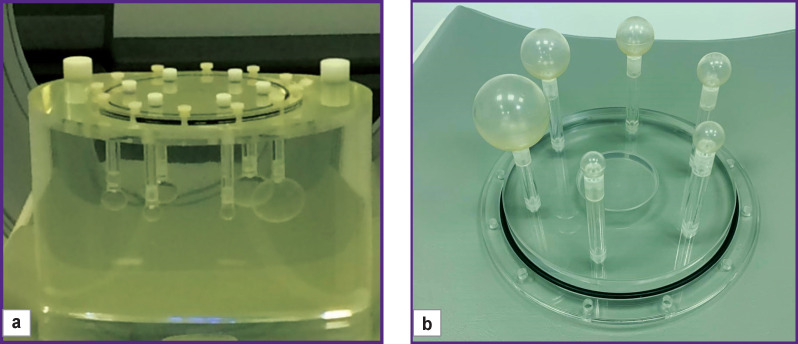
Appearance of the NEMA IEC PET Body Phantom Set (USA) and its components: (a) a main volume of the phantom with an inner length of 180 mm and a volume of 9.6 L; (b) six fillable spheres with inner diameters of 10, 13, 17, 22, 28, and 37 mm, the centers of which are located in the same plane; spheres’ wall thickness — no more than 1 mm

Preparation of a dedicated NEMA IEC PET Body Phantom Set for PET/CT scanning consisted of filling the main volume and spheres with a ^18^F solution. The appearance of the phantom and its components is shown in Figure 1.

The phantom spheres with diameters of 10, 13, 17, 22, and 28 mm were used to simulate lung lesions, the main volume of the phantom used as analogue of anatomical structures adjacent to the focus. The ^18^F activity concentration in the main volume of the phantom was lower than that in the spheres. The prepared phantom was scanned four times on each of the tested scanners: a Discovery 690 (General Electric, USA), a Biograph mCT 128 (Siemens, Germany), and a Biograph mCT 40 (Siemens) using the clinical protocols used for patients. Before each new scan, ^18^F was added to the total volume of the phantom to create a different ratio of the sphere–main volume. The analysis of the phantom images consisted of measuring radioactivity in the main volume of the phantom and in the spheres, as well as calculating the ratios of sphere–main volume. The radioactivity values in the main volume of the phantom, spheres, as well as the ratio of sphere–main volume for four scans, are presented in Table 1. The table shows that the radioactivity ratio in the spheres to the main volume at the 1^st^ scan for the selected devices was 10.3; at the 2^nd^ — 7.0; at the 3^rd^ — 3.3; at the 4^th^ — 1.7.

**Table 1:** Radioactivity in the main volume of the phantom, spheres and the sphere–main volume ratio

Part of the phantom	Scans			
	1^st^	2^nd^	3^rd^	4^th^
***Discovery 690***				
Activity concentration (kBq/ml):				
total volume	0.36	0.43	0.76	1.2
spheres	3.7	3.0	2.5	2.1
Sphere–total volume activities				
ratio	10.3	7.0	3.3	1.7
***Biograph mCT 128***				
Activity concentration (kBq/ml):				
total volume	0.40	0.46	0.79	1.3
spheres	4.0	3.2	2.6	2.2
Sphere–main volume activities ratio	10.0	7.0	3.3	1.7
***Biograph mCT 40***				
Activity concentration (kBq/ml):				
total volume	0.34	0.40	0.72	1.1
spheres	3.3	2.8	2.4	1.9
Sphere–main volume activities ratio	9.7	7.0	3.3	1.7

The parameters of the scan protocols and reconstruction, depending on the used PET/CT unit, are presented in Table 2.

**Тable 2:** Parameters of the scanning and reconstruction protocols for PET/CT scanners of different models

Parameter	Discovery 690	Biograph mCT 128	Biograph mCT 40
***Scanning parameters***			
Time per bed (min)	2.4	2.3	2.3
Scanning mode	WB	WB	WB
Bed overlapping	11	46	46
***Reconstruction parameters***			
Reconstruction method	VPFX + Sharp IR (ToF + PSF analogue)	ToF + PSF	ToF + PSF
Iteration/subset number	2/24	2/21	2/21
Reconstruction filter	Cut-Off 6.4	Hamm 5	Hamm 5
Image matrix (pixels)	192×192	256×256	256×256
Pixel size (mm)	3.64×3.64	3.18×3.18	3.18×3.18
Slice thickness (mm)	3.27	1.5	2.0

**Figure 2:**
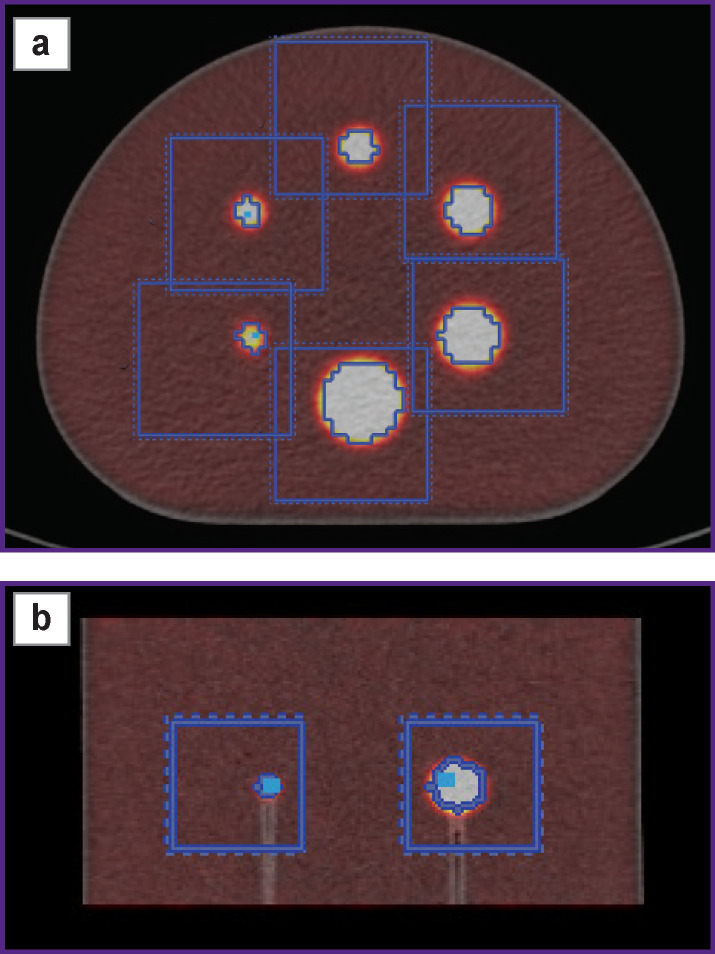
An example of delineating and measuring the maximum value of activity concentration in spheres in axial (a) and coronal (b) projections

The radioactivity of ^18^F was measured using a verified Curiementor 4 dose calibrator (PTW-Freiburg, Germany) with a relative 5% error in activity measuring. For each sphere, the volumes of interest were determined using automatic delineation in order to measure the maximum value of activity concentration. An example of delineation and measuring the maximum value of activity concentration in the spheres is shown in Figure 2.

To evaluate the reproduction of radioactivity in the lung lesions on the PET images, the RC was calculated by the formula

$$RC=\frac{A_{image}}{A_{inject}},$$

where *A*_image_ is the activity concentration in the sphere measured on PET images (kBq/ml); *A*_inject_ is the activity concentration injected into the sphere measured during preparation of the phantom for scanning (kBq/ml).

The RC values obtained for the four scans were averaged. The averaged RCs were interpolated for the unknown (intermediate) sizes of the lung lesions.

### Patient studies

The analysis of the PET/CT with ^18^F-FDG data was carried out in 86 patients with solitary newly diagnosed lung lesions: in 37 patients with malignant tumors (MT), in 49 patients with benign tumors (BT) and inflammatory diseases (ID). The study was performed in accordance with the Declaration of Helsinki (2013) and approved by the Ethics Committee of the A.M. Granov Russian Research Center for Radiology and Surgical Technologies of the Ministry of Health of the Russian Federation.

The criteria for inclusion in the study were formed on the basis of the recommendations by the Fleischner Society [12]. The following characteristics of the lung lesions at CT were required: a round shape or close to it; total size — 8–30 mm; solid or subsolid structure (with the exception of the lesions with ground-glass opacity); solid part size ≥8 mm. At the time of PET/ CT scanning, all the patients had no signs of pleurisy, lymphadenopathy, and an oncological history.

PET/CT with ^18^F-FDG was performed at the A.M. Granov Russian Research Center for Radiology and Surgical Technologies in the Department of Positron Emission Tomography on the Discovery 690, Biograph mCT 128, and Biograph mCT 40. The distribution of the patients into groups depended on the morphological diagnosis and the scanners are presented in Table 3.

**Тable 3:** Patient distribution depending on the morphological diagnosis and the scanners (abs. number/%)

Diagnosis	Discovery 690	Biograph mCT 128	Biograph mCT 40
Malignant tumors (n=37)	24/64.9	3/8.1	10/27.0
Benign tumors and inflammatory diseases (n=49)	28/57.1	8/16.3	13/26.6
Total (n=86)	52/60.5	11/12.8	23/26.7

The 60.5% of patients were scanned on a Discovery 690 scanner, 12.8% on a Biograph mCT 128 scanner, and 26.7% on a Biograph mCT 40 scanner. The preparation of patients prior to the scan was standardized. The study was limited to scanning one anatomical region — the thoracic region — and started 70–90 min after intravenous administration of ^18^F-FDG with the activity of 110 MBq per unit of the patient’s body surface area. The scan protocol consisted of a topogram, helical CT scan without contrast enhancement for attenuation correction, and PET scan.

The post-processing data analysis consisted of visual evaluation of CT, PET, and hybrid images, as well as performing a semi-quantitative analysis. The measurement of SUVs was performed by automatic delineation of the volume of interest (VOI) in a specialized program on an AW 4.7 workstation (General Electric). The SUV_max_ normalized to lean (muscle) body mass (SUL) were considered to be diagnostically significant levels of radiopharmaceutical uptake in the lung lesions. SUV_max_ (SUL) calculation was performed by the software package automatically according to the formula

$$SUV_{\max}(SUL)=\frac{A_{VOI}}{A_{inject}/LBM},$$

where *A*_VOI_ is the radioactivity in the volume of interest (MBq/ml); *A*_inject_ is the total injected activity to the patient, corrected for lean (muscle) body mass (MBq/kg); LBM — lean body mass.

The corrected maximum SUVs (SUV_correct_) were calculated by the formula

$$SUV_{correct}=\frac{SUV_{\max}}{RC},$$

where SUV_max_ is the maximum level of radiopharmaceutical uptake in the lung lesions, on the PET image; RC is the ratio of the activity concentration in the sphere of the phantom, to the activity concentration injected into the sphere during the preparation of the phantom for scanning.

### Statistical data processing.

The data analysis was performed applying the MedCalc v. 19.2.0 software. The distribution was checked for normality using the Kolmogorov–Smirnov test (corrected for the Lilliefors). Applying the methods of descriptive statistics, the median and 95% confidence interval (CI) were calculated. The statistical significance of the differences between the values was calculated using the Wilcoxon test. The critical level of statistical significance of the null statistical hypothesis was taken equal to 0.05. Spearman’s correlation coefficient (ρ) was calculated to study the relationship between the variables. The qualitative characteristic of the relationship between the studied variables was assessed using the Chaddock scale (0.10–0.30 — a weak relationship; 0.31–0.50 — a moderate relationship; 0.51–0.70 — a noticeable relationship; 0.71–0.90 is a high relationship; 0.91–1.0 is a very high relationship). For visual data representation, the box and whiskers plots were used.

## Results

### Phantom studies

The RCs, averaged for dilutions and interpolated for unknown (intermediate) lesion sizes in the lungs, for four scans of the NEMA IEC PET Body Phantom Set with different ^18^F solution on three scanners, are presented in Table 4. The table demonstrates that the RCs varied relative to the value of 1.0 on all the scanners with different sizes of lesion. The RCs <1.0 indicated the underestimation of the radiopharmaceutical accumulation levels; the RCs >1.0 showed their overestimation; the RCs=1 demonstrated accordance with the radiopharmaceutical accumulation levels with the reference values.

**Table 4:** RCs averaged by four scans and interpolated for unknown (intermediate) lung lesion sizes of the NEMA IEC PET Body Phantom Set

Diameter (mm)	Recovery coefficients		
	Biograph mCT 128	Biograph mCT 40	Discovery 690
30	1.22	1.20	1.05
29	1.20	1.19	1.04
28	1.19	1.18	1.03
27	1.16	1.17	1.02
26	1.14	1.16	1.0
25	1.12	1.15	0.99
24	1.09	1.14	0.98
23	1.07	1.13	0.97
22	1.04	1.12	0.95
21	1.02	1.10	0.92
20	0.99	1.08	0.89
19	0.97	1.06	0.86
18	0.94	1.04	0.83
17	0.92	1.02	0.80
16	0.89	0.98	0.77
15	0.86	0.94	0.73
14	0.83	0.90	0.70
13	0.80	0.87	0.66
12	0.72	0.77	0.57
11	0.64	0.67	0.48
10	0.56	0.58	0.39
9	0.49	0.48	0.30
8	0.46	0.38	0.27

### Patient studies

**Figure 3:**
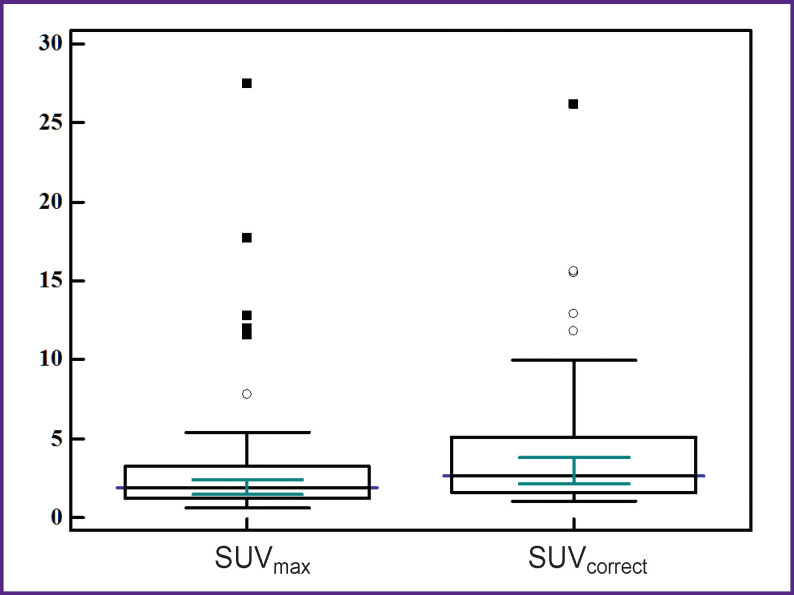
The range of SUV_max_ and SUV_correct_ in the patients with malignant tumors in the lungs

In 37 patients with MT, the sizes of the detected lung lesions on the CT images ranged from 8 to 30 mm (median — 16.5; 95% CI 13.0–18.0). A direct correlation between the lesion size and SUV_max_ was found (ρ=0.59; 95% CI 0.33–0.77; p=0.0001). No correlation was found between the size of the lesions and SUV_correct_ (ρ=0.24; 95% CI 0.09–0.53; p=0.1546). The SUV_max_ and SUV_correct_ are shown in Figure 3.

The values in the box plots show the minimum and maximum values, 25^th^ and 75^th^ percentiles, and medians with 95% CI. The outliers were considered to be the values lying in the ranges exceeding the height of the box from its upper and lower boundaries by 1.5 times.

Figure 3 shows that SUV_max_ in patients with MT had a wide range (from 0.6 to 27.5), differed among themselves significantly (p˂0.0001); the median was determined at the level of 1.9 (95% CI 1.5–2.4). After applying the RC, the corrected levels of radiopharmaceutical accumulation were determined within the range of 1.1–26.2, the median SUV_correct_ was 2.6 (95% CI 2.1–3.8). No statistically significant differences were found (p=0.0024) between SUV_max_ and SUV_correct_.

**Figure 4:**
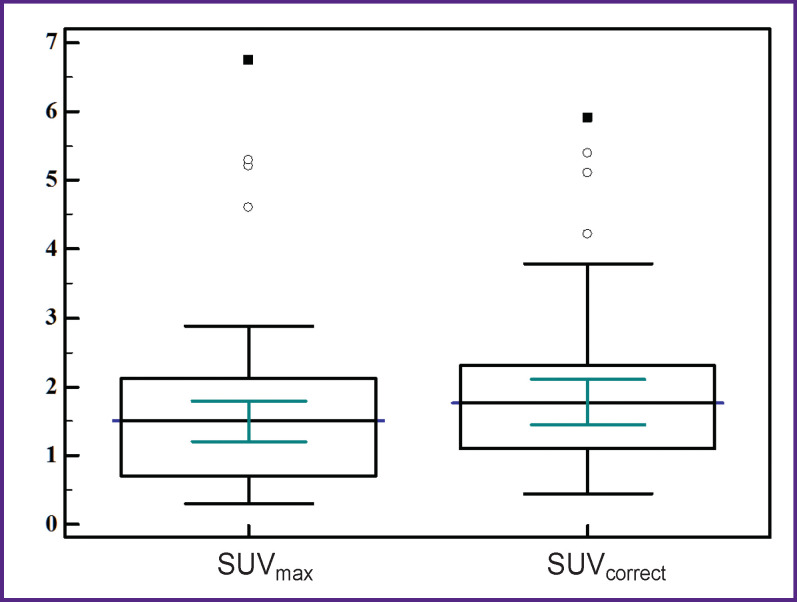
The range of SUV_max_ and SUV_correct_ values in the patients with benign tumors and inflammatory lung disease

In 49 patients with BT and ID the size of the detected lesions on the CT images varied from 8 to 29 mm, the median was 16.0 (95% CI 14.0–18.8). When comparing the size of the lesion and the SUV_max_, a direct high correlation was found between these characteristics (ρ=0.61; 95% CI 0.39–0.76; p<0.0001). No correlation was found between the lesion size and SUV_correct_ (ρ=0.19; 95% CI 0.09–0.45; p=0.1872). The SUV_max_ and SUV_correct_ recorded in the patients with BT and ID are shown in Figure 4.

The values in the box plots show the minimum and maximum values, 25^th^ and 75^th^ percentiles, and medians with 95% CI. The outlying values were considered those

Figure 4 shows that the SUV_max_ in the patients with BT and ID were determined in the range of 0.3–6.7; the median was 1.5 (95% CI 1.2–1.8). After applying the RC, the corrected levels of radiopharmaceutical accumulation ranged from 0.5 to 5.9; the median was 1.8 (95% CI 1.5–2.1). The comparison of SUV_max_ and SUV_correct_ showed statistically significant differences (p=0.0006).

Table 5 shows the frequency distribution of the examined patients, depending on the RC value. The table shows that the overwhelming number of the patients, regardless of the morphological diagnosis, showed a distortion of the levels of radiopharmaceutical accumulation. Most often (73.2%), the underestimation of SUV_max_ was recorded in the lesions from 10 to 20 mm. The SUV_max_ overestimation was observed in 25.6% of the patients with larger lesions (20–28 mm). Only in one case, the measured level of radiopharmaceutical uptake in the tumor corresponded to the reference value in a patient with MT scanned on the PET/CT Discovery 690. According to the phantom studies, the RC on this scanner equals 1 when the focus size is 26 mm.

**Тable 5:** Distribution of the examined patients according to the RC

Diagnosis	RC <1.0			RC=1.0			RC >1.0		
	Number of patients (abs. number/%)	Lesion size (mm) [median (95% CI)]	SUV_max_ [median (95% CI)]	Number of patients (abs. number/%)	Lesion size (mm) [median (95% CI)]	SUV_max_ [median (95% CI)]	Number of patients (abs. number/%)	Lesion size (mm) [median (95% CI)]	SUV_max_ [median (95% CI)]
Malignant tumors (n=37)	28/75.7	14.2 (11–17)	1.5 (1.2–1.9)	1/2.7	26	11.6	8/21.6	23.5 (18.0–29.2)	6.3 (2.6–20.0)
Benign tumors and inflammatory diseases (n=49)	35/71.4	14.5 (12–15)	1.2 (0.7–1.5)	—	—	—	14/28.6	24 (21–26)	2.1 (1.5–3.1)
Total (n=86)	63/73.2	14.4 (13–15)	1.8 (1.1–1.5)	1/1.2	—	—	22/25.6	24 (21–26)	2.7 (1.9–5.2)

**Figure 5:**
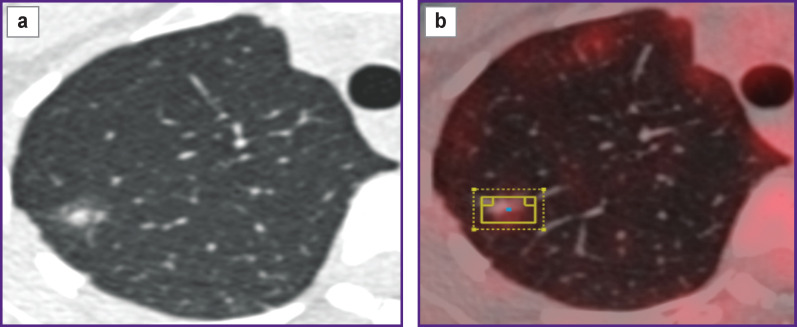
Diagnosis: “acinar adenocarcinoma of the upper lobe of the right lung; G2; pT1aN0cM0; IA”. The study was performed on a PET/CT Discovery 690: (a) CT scan in the S3 segment of the right lung detects a subsolid lesion with the total size 12 and 8 mm of the solid part; (b) PET detects a lesion that minimally accumulates radiopharmaceutical; SUV_max_=0.9; RC=0.27; SUV_correct_=3.5

It is important to note the clinical significance of the obtained findings. A considerable underestimation of SUV_max_ nearly leads to obtaining false negative results in 9 MT patients (32.1%) with the lung lesions ranged from 9 to 12 mm. In all these cases, due to the low level of radiopharmaceutical uptake in the MT, the changes in the lung were erroneously interpreted as an inflammatory process. lying in the ranges exceeding the height of the box from its upper and lower boundaries by 1.5 times. Applying RC in these patients ensured objective assessment of the radiopharmaceutical accumulation levels and, as a consequence, correctly suggested the nature of the tumor (Figure 5).

Thus, the results of the phantom and patient studies show that, regardless of the used scanners in the lesions up to 20 mm during PET/CT scanning, a considerable underestimation of SUV_max_ is observed. It might lead to underestimation of the biological aggressiveness of a tumor and, as a consequence, an increase in the number of false negative results. At the same time, with larger lesion sizes, depending on the scanner model, SUV_max_ overestimation may be observed. All these distortions in the value measurements in the routine practice of PET departments must undoubtedly be corrected.

## Discussion

Currently, PET/CT is one of the principal methods of diagnostics and evaluation of the treatment effectiveness of various oncological diseases. The given technology is characterized by substantially high visualizing capabilities and, at the same time, is a qualitative method that allows for measuring various biochemical processes ongoing in the human body. To solve clinical problems with the utility of PET/CT as quantitative criteria, different indicators are used: SUV_max_, metabolic tumor volume (MTV), total lesion glycolysis (TLG), average ratio of the tumor-to-background radioactivity (TBR), standardized uptake peak value determined within the VOI of the fixed size (SUV_peak_), etc. [13–18]. The most common one among them is SUV_max_, which is determined by the semiquantitative method [15].

SUV displays the intensity of radiopharmaceutical accumulation in the target volume of interest and depends on the volume in which the given activity is spread [15]. There are several techniques of calculating SUVs depending on the patient’s body mass, surface area of the body, as well as SUL [15, 19, 20]. In compliance with the latest recommendations by the European Association of Nuclear Medicine (EANM), the definition of SUL. At the same time, it is important to note that SUV_max_, as well as other quantitative values, depends on several factors: a glucose level in the patient’s blood plasma, duration of radiopharmaceutical accumulation, parameters of the scan protocol, data reconstruction algorithms, accuracy of volume of interest determination and delineation method, intensity of radiopharmaceutical accumulation in the adjoining tissues, heterogeneity of a lesion structure, etc [1–7].

Another important factor that affects both the qualitative and quantitative characteristics of PET images is PVE [5–7]. This effect is based on the existing limitations in the spatial resolution of PET modality, which lead to unsatisfactory visualization of small tumors and underestimation of SUV_max_ relative to the true levels of radiopharmaceutical accumulation. Because of PVE, the lesions with the same level of radiopharmaceutical accumulation, but differing size and histological origin, are displayed on PET images with different brightness. As a result, tumors of a malignant nature may be erroneously regarded as less aggressive, and this leads to an increase in the number of false negative results at PET/CT with ^18^F-FDG.

Today, the most famous and easily reproducible method for PVE correction is the use of RC, which is determined on the basis of the phantom studies [15, 21]. According to the obtained data, the RC becomes close to 1.0 with the increase in lesion size. In other words, the small lesions are more affected the underestimation of radiopharmaceutical accumulation. Thus, the negative effect of PVE in the lesion with a diameter of 8 mm led to SUV_max_ underestimation from 54 to 73% for different scanners. At the same time, in 9 patients having MT with the size of the pathological lung lesions ranged from 9 to 12 mm, the use of RC avoids false negative results. In all these cases, the MT was not clearly visible on the background of an intact lung, and the SUV_max_ for the lesion did not exceed 1.0.

According to the literature [6, 15, 21], the impact of PVE becomes more detectable for the lung lesions located near the anatomical structures, which are normally characterized by an increased radiopharmaceutical accumulation. Usually, at PET/CT with ^18^F-FDG imaging, physiological radiopharmaceutical pathological accumulation is observed in the mediastinum, liver, left ventricular myocardium, and ribs. According to these studies, RC may be ineffective for the tumors located close to at least two anatomical structures with different radiopharmaceutical accumulation.

The severity of the PVE also depends on the shape of the tumor. It is generally assumed that PVE is more pronounced for larger the surface area of the pathological lesions [6, 22]. Accordingly, tumors that have a spherical, i.e., more compact form, are less susceptible to a negative impact of PVE. This is due to the fact that in the lesions of an irregular shape and/or heterogeneous structure the zone of necrotic softening is located in the center and volume with actively accumulates radiopharmaceutical is located mainly in the peripheral parts of the tumor. In these cases, intensive processes of radioactivity “spill-over” between the periphery of the tumor, adjoining anatomical structures and the necrotic center of the tumor, determine the strong influence of PVE on the quantitative PET characteristics.

On the other hand, the pathological lesions with the diameter of 30 mm showed a marked SUV_max_ overestimation — by 22%. According to the literature [1–3, 16, 23, 24], SUVs overestimation is connected with reconstruction algorithms, such as PSF and ToF. Both algorithms are used in modern hybrid scanners to increase the sensitivity of the PET/CT method, improving the signal/noise ratio and reducing the scan duration. As shown in the given study, the negative influence of the reconstruction methods on SUV_max_ in the lesion of a larger size can also be eliminated with the RC. Correction of SUVs in these cases can prevent obtaining false positive results.

Direct correlation between SUV_max_ and the sizes of the lung lesions was confirmed by the results of numerous studies [25–28]. In the course of the given study, the statistical correlation between these criteria was found in the patients of both groups. It should be noted that, after applying RC, the statistical correlation between the lesion sizes and SUV_correct_ was not determined for all morphological diagnoses and scanners. It evidenced that RC eliminated a negative impact of PVE on SUV_max_ in the small lesions and also showed the influence of the reconstruction methods on the larger lesions.

In 2010, the EANM launched a accreditation programme (European Association Research Limited, EARL) of the medical facilities performing PET/CT with ^18^F-FDG [2, 10, 23, 28–31]. This strategy was aimed at minimization of variability of qualitative PET-criteria by standardization of the data acquisition and reconstruction protocols. Currently, the EARL programme validation is described as the evaluation of the effectiveness of treatment of MT in various tissues including lung cancer. By 2016, the Clinical Trials Network (CTN) collected in the accredited EANM–EARL system the results of 2500 phantom studies were obtained approximately in 200 scanners of different models in 150 medical facilities around the world [10].

Today, professional societies as the American College of Radiology Imaging Network (ACRIN), the Radiologic Society of North America’s Quantitative Imaging Biomarker Alliance, American Association of Physicists in Medicine are also actively working to promote the concept of harmonization of PET/CT [30]. In our country, this area has just begun to develop. In 2020, Rospotrebnadzor published the methodological guidelines for optimization of the quality control procedures and stability of PET image parameters using the phantom [11].

## Conclusion

The use of the recovery coefficient neutralizes the PVE and impact of the reconstruction methods on the accuracy of SUV_max_ in the lung lesions, which allows for increasing the information of the PET/CT with ^18^F-FDG method and ensures the reproducibility and comparability of the results obtained on the scanners of different models with different technological characteristics.
